# Interpreting the PISCES trial on fish oil in hemodialysis patients: lessons on trial design, biological exposure, and outcome measurement

**DOI:** 10.1093/ckj/sfag118

**Published:** 2026-04-16

**Authors:** Jaime Mazón-Ruiz, Marina de Cos Gomez, Paula González Bores, Pablo Corral

**Affiliations:** Nephrology Department, Hospital Carmen y Severo Ochoa, Cangas del Narcea, Spain; Nephrology Department, Hospital Universitario Central de Asturias, Oviedo, Spain; Department of Medicine, University of Oviedo; Division of Nephrology, Department of Medicine, Icahn School of Medicine at Mount Sinai, New York, NY, USA; Department of Internal Medicine. Hospital Universitario de Sierrallana, Torrelavega, Spain; Department of Pharmacology, Faculty of Medicine, Universidad FASTA, Clinical Researcher at the Clinical Research Institute (IIC), Mar de Plata, Argentina

**Keywords:** cardiovascular outcomes, hemodialysis, icosapent ethyl, omega-3 fatty acids, recurrent events

## Abstract

Patients receiving maintenance hemodialysis experience an exceptionally high cardiovascular risk, characterized by frequent recurrent events and a longstanding lack of effective preventive therapies. Most cardiovascular trials in this population have yielded neutral results, highlighting important limitations in both therapeutic strategies and outcome assessment.

The PISCES trial recently reported significant reductions in both first and recurrent cardiovascular events with high-dose omega-3 fatty acids in patients undergoing hemodialysis, using a recurrent-event framework to capture overall cardiovascular burden. In this focused narrative review, we critically appraise the PISCES findings within the broader context of omega-3 research in chronic kidney disease and dialysis. Particular emphasis is placed on how biological exposure, population selection, and outcome frameworks influence the detectability of cardiovascular benefit in high-risk populations.

PISCES challenges the prevailing notion that cardiovascular risk in hemodialysis is largely unmodifiable and underscores the importance of aligning therapeutic interventions with outcome measures that reflect total disease burden. More broadly, the trial illustrates how methodological choices may critically influence the interpretation of cardiovascular efficacy in populations with dense event clustering and high competing mortality.

## GRAPHICAL ABSTRACT

**GRAPHICAL ABSTRACT:**
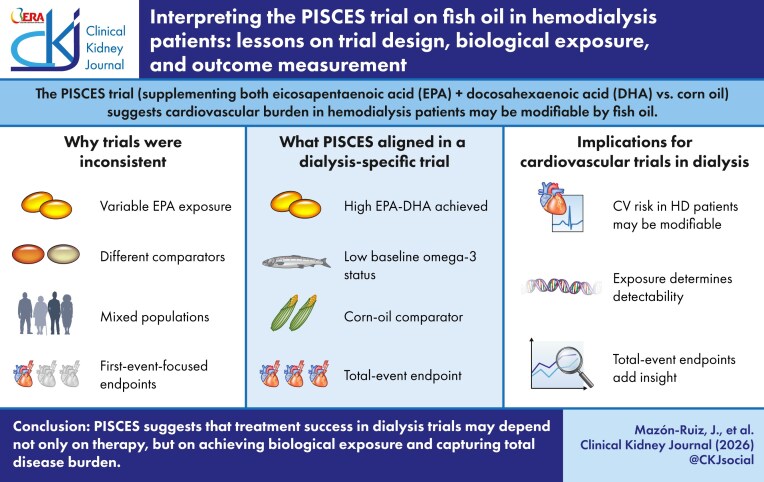

## INTRODUCTION

For more than two decades, large, randomized trials have repeatedly failed to demonstrate a clear cardiovascular benefit of therapies in patients receiving maintenance hemodialysis. In this discouraging landscape, the PISCES trial represents a rare and provocative signal of benefit. It reports a substantial reduction in cardiovascular event burden with a fish-oil formulation containing both eicosapentaenoic acid (EPA) and docosahexaenoic acid (DHA) in dialysis patients. The accompanying editorial in *New England Journal of Medicine* described these findings as “remarkable but unexpected,” urging confirmation before changes in clinical practice [1, 2].

Rather than reassessing omega-3 therapy itself, this editorial uses PISCES as a case study to explore how biological exposure, comparator choice, and outcome measurement together shape the visibility of cardiovascular benefit in dialysis populations.

## UNDERSTANDING THE HETEROGENEITY OF PRIOR OMEGA-3 CARDIOVASCULAR TRIALS

Prior omega-3 cardiovascular trials have yielded heterogeneous and sometimes conflicting results (Table 1). While some studies demonstrated reductions in cardiovascular events, others reported neutral findings, and several meta-analyses have likewise produced divergent conclusions [3–5].

**Table 1:** Major randomized cardiovascular outcome trials of omega-3 fatty acids.

Trial	Clinical population	Omega-3 formulation (dose/day)	Comparator	Follow-up (years)	Primary endpoint framework	Main primary result
GISSI-Prevenzione	Prior MI	EPA 290 mg + DHA 580 mg	Usual care	3.5	Composite CV endpoint (death, non-fatal MI, stroke)	Reduction in CV death and composite endpoint
GISSI-HF	Symptomatic HF	EPA 290 mg + DHA 580 mg	Placebo	3.9	All-cause death or CV hospitalization	Small but significant reduction in mortality and hospitalization
Alpha Omega	Prior MI	EPA 226 mg + DHA 150 mg	Placebo margarine	3.4	Composite CV endpoint (CV death, MI, stroke, revascularization)	No significant reduction in major cardiovascular events
ORIGIN	Diabetes or dysglycemia	EPA 465 mg + DHA 375 mg	Olive oil	6.2	Three-point MACE^a^	No reduction in MACE
ASCEND	Diabetes without ASCVD	EPA 460 mg + DHA 380 mg	Olive oil	7.4	First serious vascular events	No significant reduction in serious vascular events
VITAL	General population	EPA 460 mg + DHA 380 mg	Placebo	5.3	Three-point MACE^a^	No significant reduction in MACE
STRENGTH	High CV risk with dyslipidemia	EPA 2.2 g + DHA 0.8 g	Corn oil	3.5	Four-point MACE^b^	No significant reduction in MACE
JELIS	Hypercholesterolemia on statin therapy	EPA 1.8 g	Statin alone	4.6	Composite CV endpoint (sudden death, MI, unstable angina, revascularization)	Reduction in CV composite
REDUCE-IT	Residual hypertriglyceridemia on statin therapy	EPA 4 g	Mineral oil	4.9	Composite CV endpoint (CV death, MI, stroke, revascularization, unstable angina)	Reduction in CV composite
PISCES	Hemodialysis	EPA 1.6 g + DHA 0.8 g	Corn oil	3.5	Total CV events (first + recurrent)	Significant reduction in total CV events and cardiac mortality

ASCVD, atherosclerosis cardiovascular disease; HF, heart failure; MACE, major adverse cardiovascular events; MI, myocardial infarction.

a Three-point MACE: CV death, MI, stroke.

b Four-point MACE: CV death, MI, stroke, unstable angina.

Dose and formulation represent one potential explanation. Many early trials of mixed EPA + DHA formulations used relatively modest doses, whereas studies demonstrating benefit—including JELIS and REDUCE-IT—achieved substantially higher EPA exposure [6, 7].

Population differences also contribute to heterogeneity. Most prior omega-3 outcome trials enrolled patients with established atherosclerotic cardiovascular disease or high cardiovascular risk, but none specifically targeted patients receiving dialysis. This limits extrapolation to dialysis populations, who not only exhibit distinct cardiovascular pathophysiology but also tend to have lower baseline circulating omega-3 levels than the general population [8, 9].

Biological exposure represents another critical but often overlooked factor. Trials showing benefit typically documented substantial increases in circulating or erythrocyte omega-3 levels, whereas many neutral studies did not systematically assess achieved fatty-acid incorporation [5].

Beyond exposure, omega-3 benefits may extend beyond lipid modification. REDUCE-IT demonstrated that cardiovascular risk reduction with EPA was consistent in patients with triglyceride levels above and below 150 mg/dl [7], supporting mechanisms such as anti-inflammatory activity, membrane stabilization, and modulation of arrhythmic risk.

Comparator choice further complicates interpretation. In REDUCE-IT, the mineral-oil placebo was associated with modest increases in low-density lipoprotein cholesterol, apoB, and inflammatory biomarkers [10, 11], raising concerns that part of the observed treatment effect could reflect comparator-related harm rather than pure benefit. Although subsequent analyses suggest that these changes explain only a fraction of the effect, the episode illustrates how placebo selection can influence apparent efficacy. Importantly, these considerations do not replace standard lipid management.

BOX 1. PISCES at a glance.

**Table:** 

Design: multicenter, randomized, double-blind, placebo-controlled trial in hemodialysis
Population: 1228 adults receiving chronic hemodialysis
Intervention: fish-oil formulation (EPA 1.6 g/day + DHA 0.8 g/day) vs corn-oil placebo
Follow-up: median 3.5 years
Primary endpoint: major cardiovascular (CV) events (CV death, non-fatal MI, non-fatal stroke, hospitalization for heart failure, or clinically significant arrhythmia) analyzed as first and recurrent eventsEvent rate: fish oil: 0.31 per 1000 patient-days vs placebo: 0.61 per 1000 patient-days{hazard ratio (HR) 0.57 [95% confidence interval (CI) 0.47–0.70]}
First CV event or death: HR 0.73 (95% CI 0.61–0.87)
Total CV events: HR 0.57 (95% CI 0.47–0.70)
Proportion of patients experiencing more than or equal to two events: 4.3% in the fish-oil group vs 10.5% in the placebo group
Mortality outcomesCardiac death: HR 0.55 (95% CI 0.40–0.75)All-cause mortality: HR 0.89 (95% CI 0.73–1.01)

## METHODOLOGICAL INSIGHT: FIRST VERSUS RECURRENT EVENTS

First events, recurrent non-fatal events, and terminal outcomes capture distinct dimensions of disease. First-event analyses reflect the timing of the initial clinical manifestation, recurrent-event frameworks quantify cumulative morbidity, and terminal outcomes reflect ultimate prognosis. These endpoints should therefore be interpreted as complementary rather than interchangeable.

Recurrent-event approaches provide additional insight by capturing the total burden of cardiovascular complications over time. However, reductions in recurrent events do not necessarily translate into improved survival. In high-risk populations such as those receiving dialysis, benefits may reflect delayed progression, reduced clustering of complications, or changes in management after initial events rather than changes in ultimate prognosis.

A *post-hoc* analysis of the 4D statin trial illustrates this nuance. While the primary first-event endpoint was neutral, recurrent-event modeling suggested fewer subsequent events among treated patients, without a clear mortality effect [12].

REDUCE-IT reached broadly similar conclusions. Icosapent ethyl reduced both first and subsequent ischemic events, lowering overall cardiovascular burden, yet effects on mortality were modest [7].

PISCES extends these observations to the dialysis population. The consistency of its findings across several recurrent-event models—including Andersen–Gill, Prentice–Williams–Peterson, and shared-frailty approaches—suggests that the signal is not dependent on a single statistical framework. Andersen–Gill treats events as a continuous process, Prentice–Williams–Peterson accounts for event ordering, and frailty models account for individual susceptibility. Nevertheless, consistency across models does not eliminate potential biases related to competing mortality, outcome ascertainment, or clustering of events in high-risk individuals. First-event analyses therefore remain clinically relevant even in populations with limited survival.

## SAFETY AND COMPARATOR CONSIDERATIONS

In PISCES, overall mortality did not differ between groups, but cardiovascular deaths were reduced. Minor bleeding events were slightly more frequent with fish oil, consistent with prior experience with high-dose EPA, yet major bleeding was not increased. The corn-oil comparator is generally considered less likely than mineral oil to influence lipid or inflammatory profiles, although it should not be assumed biologically inert. This nuance is important when interpreting treatment effects in contemporary cardiovascular trials.

## WHY PISCES MATTERS

PISCES should not be interpreted as a definitive positive trial and its findings will require validation, but it raises the possibility that important lessons can be drawn for the design and interpretation of cardiovascular trials in dialysis populations. Rather than representing an isolated signal, PISCES can be viewed as the convergence of several factors previously identified as potential sources of heterogeneity in omega-3 cardiovascular research. Prior studies differed in achieved omega-3 exposure, comparator selection, study populations, and outcome measurement.

PISCES aligns these dimensions within a single trial: it achieved relatively high omega-3 exposure, employed a comparator considered less controversial than mineral oil, enrolled a population with marked cardiovascular vulnerability and typically low baseline omega-3 status, incorporated measurement of circulating omega-3 levels in a subset of participants, and adopted an endpoint designed to capture cumulative cardiovascular burden rather than isolated first events.

From this perspective, PISCES may be viewed less as an isolated positive trial and more as an example in which several methodological and biological conditions previously discussed in the literature were jointly optimized within the dialysis setting.

## CLINICAL IMPLICATIONS

PISCES provides some of the strongest contemporary evidence that cardiovascular burden in dialysis populations may be modifiable. The trial demonstrated reductions in both first and recurrent cardiovascular events and a decrease in cardiac mortality, although overall survival was not significantly improved. This pattern suggests that omega-3 therapy may meaningfully reduce cardiovascular burden in dialysis populations, even if its long-term impact on survival remains uncertain.

At present, regulatory confirmation and replication in independent cohorts remain important before universal adoption. However, the findings challenge the long-standing assumption that pharmacologic cardiovascular prevention is largely ineffective in dialysis populations and raise the question of whether carefully selected high-risk patients could potentially benefit from omega-3 therapy, given its favorable safety profile and pending further confirmation of efficacy.

If omega-3 therapy is contemplated in such contexts, several principles may help guide its use:

• Prefer formulations achieving substantial EPA exposure (≥1.6 g/day or EPA: DHA ≥ 2:1).
• Integrate therapy within multifactorial cardiovascular prevention strategies.
• Monitor for atrial fibrillation, infection, and bleeding, although serious events appear to remain uncommon.

Rather than definitively establishing a new standard of care, PISCES reframes the clinical question: Under what biological, pharmacologic, and methodological conditions can cardiovascular benefit be achieved in dialysis populations?

## CONCLUSIONS AND FUTURE PERSPECTIVES

PISCES represents a potentially important advance in the search for cardiovascular protection in dialysis populations. Beyond its specific therapeutic findings, the trial illustrates how cardiovascular benefit in this setting may depend not only on the intervention itself but also on biological targeting and exposure as well as trial design and endpoint selection.

These insights extend beyond omega-3 research and may inform the evaluation of other cardiovascular interventions in hemodialysis populations, where high event density and competing mortality risks often obscure treatment effects.

Replication and further mechanistic study remain warranted, particularly to clarify exposure–response relationships and identify patients most likely to benefit. However, PISCES has reopened a therapeutic space long considered unmodifiable and suggests that both biological targeting and methodological rigor will determine whether future cardiovascular therapies can demonstrate benefit in dialysis populations.

## Data Availability

No new data were generated or analyzed during the current study.
